# Short‐term Sahaja Yoga meditation training modulates brain structure and spontaneous activity in the executive control network

**DOI:** 10.1002/brb3.1159

**Published:** 2018-11-28

**Authors:** Alessandra Dodich, Maurizio Zollo, Chiara Crespi, Stefano F. Cappa, Daniella Laureiro Martinez, Andrea Falini, Nicola Canessa

**Affiliations:** ^1^ Division of Neuroscience San Raffaele Scientific Institute Milan Italy; ^2^ NIMTlab University of Geneva Geneva Switzerland; ^3^ Invernizzi Center for Research in Innovation, Organization and Strategy Bocconi University Milan Italy; ^4^ NeTS Center Scuola Universitaria Superiore IUSS Pavia Italy; ^5^ IRCCS San Giovanni di Dio Fatebenefratelli Brescia Italy; ^6^ MTEC ETH Zurich Switzerland; ^7^ Neuroradiology Unit San Raffaele Scientific Institute Milan Italy; ^8^ Cognitive Neuroscience Laboratory ICS Maugeri Pavia Italy

**Keywords:** fronto‐parietal executive control network, neural plasticity, resting‐state *f*MRI, Sahaja Yoga meditation, voxel‐based morphometry

## Abstract

**Introduction:**

While cross‐sectional studies have shown neural changes in long‐term meditators, they might be confounded by self‐selection and potential baseline differences between meditators and non meditators. Prospective longitudinal studies of the effects of meditation in naïve subjects are more conclusive with respect to causal inferences, but related evidence is so far limited.

**Methods:**

Here, we assessed the effects of a 4‐week Sahaja Yoga meditation training on gray matter density and spontaneous resting‐state brain activity in a group of 12 meditation‐naïve healthy adults.

**Results:**

Compared with 30 control subjects, the participants to meditation training showed increased gray matter density and changes in the coherence of intrinsic brain activity in two adjacent regions of the right inferior frontal gyrus encompassing the anterior component of the executive control network. Both these measures correlated with self‐reported well‐being scores in the meditation group.

**Conclusions:**

The significant impact of a brief meditation training on brain regions associated with attention, self‐control, and self‐awareness may reflect the engagement of cognitive control skills in searching for a state of mental silence, a distinctive feature of Sahaja Yoga meditation. The manifold implications of these findings involve both managerial and rehabilitative settings concerned with well‐being and emotional state in normal and pathological conditions.

## 1 INTRODUCTION

Research over the past two decades has started to unveil the neural modifications associated with different types of meditation, resulting in beneficial effects on emotional balance and cognitive performance (see Dahl, Lutz, & Davidson, 2015; Sedlmeier et al., 2012). Several studies based on cross‐sectional comparisons suggest that the effects of long‐term meditation practice may result in neuro‐anatomical and functional changes. Despite differences in subject characteristics and type of meditation, these studies highlighted significant changes in fronto‐insular, anterior cingulate, and hippocampal cortex, regions related to, respectively, self‐awareness, self‐regulation, and episodic memory (see (Fox et al., 1995) for a review; (Villemure, Ceko, Cotton, & Bushnell, 2014)). Other studies addressed the effects of extensive meditation training on intrinsic (task‐free) brain activity (Jang et al., 2011; Taylor et al., 2013), that is, the low‐frequency fluctuations of BOLD signal (<0.1 Hz) observed at rest in networks overlapping the typical task‐related activation maps (Smith et al., 2009). However, the cross‐sectional nature of these studies does not allow to establish a causal relationship between meditation practice and such changes.

To overcome this limitation, longitudinal studies assessed the effect of meditative interventions compared with a test–retest control group. Their results suggest that short‐term meditation practice may induce significant morphologic changes via mechanisms of structural plasticity (Holzel et al., 2011; Santarnecchi et al., 2014). To investigate training‐related changes in functional connectivity during mindful rest, Kilpatrick and colleagues (Kilpatrick et al., 2011) employed a group independent component analysis (gICA), a data‐driven multivariate approach highlighting subtle interindividual differences in intrinsic brain functioning (Koch et al., 2010). Subjects were explicitly instructed to be mindfully aware of the MR scanner sounds, and the results showed increased functional connectivity of the auditory cortex with brain regions underlying attentional and self‐referential mechanisms.

Based on their targeted cognitive processes, meditation practices can be grouped into two broad categories: focused attention (i.e., concentrative) and open monitoring meditation (Lutz, Slagter, Dunne, & Davidson, 2008). In concentrative practices, subjects develop regulative skills of selective attention, while in open monitoring meditation a greater emphasis is placed on cultivating a “reflexive” awareness. It is thus likely that these two training procedures may exert different effects on brain activity and/or structure (Travis & Shear, 2010).

Among open monitoring practices, Sahaja Yoga (SY) meditation is a technique based on the attempt to obtain the state of mental silence (i.e., free from unnecessary mental activity), during which all the attention is on the present moment (Aftanas & Golocheikine, 2001; Hernandez, Suero, Barros, Gonzalez‐Mora, & Rubia, 2015; Hernandez, Suero, Rubia, & Gonzalez‐Mora, 2007; Reva, Pavlov, Loktev, Korenyok, & Aftanas, 2014). With practice, the short‐term interruption of mental activity can evolve into an enduring absence of narrative thought. This mental state of “thoughtless awareness” is associated with the experience of joy, a subsequent sense of relaxation and positive mood, as well as an increase in self‐awareness (Aftanas & Golocheikine, 2001). SY differs from a concentrative type of meditation because it involves a receptive, non judgmental disposition toward all experiences, irrespective of their origin (i.e., external or internal) and affective tone (Reva et al., 2014). Previous studies reported preliminary evidence of positive effects of SY meditation in disorders such as asthma (Manocha, Marks, Kenchington, Peters, & Salome, 2002), epilepsy (Panjwani et al., 2000), attention‐deficit/hyperactivity disorder (Harrison, Manocha, & Rubia, 2016), anxiety, and work stress (Manocha, Black, Sarris, & Stough, 2011). The long‐term effects of SY meditation on neural activity have been examined by cross‐sectional studies on expert meditators with electroencephalography (EEG) (Aftanas & Golocheikine, 2001, 2002; Aftanas & Golosheikin, 2003) and functional magnetic resonance imaging (*f*MRI) (Hernandez et al., 2007). Their results suggest that SY meditation might promote functional changes in inferior frontal, parietal, and temporal regions associated with sustained attention, self‐control, and self‐awareness (Hernandez et al., 2007), possibly reflecting increased awareness of sensory stimuli and attentional control to the present moment. To the best of our knowledge, only one cross‐sectional study investigated in expert meditators the long‐term effects of SY meditation on brain structure (Hernandez et al., 2015). Compared with novices, meditators displayed increased gray matter (GM) volume in several regions, mostly in the right hemisphere, associated with sustained attention, compassion, and interoceptive perception. Namely, neurostructural changes in the right fronto‐insular and inferior temporal cortex were suggestive of neural plasticity associated with regular practice of this meditation. To date, however, no prospective study has tested the causal effect of short‐term SY meditation practice on brain structure and/or function. We aimed to fill this gap with a longitudinal randomized controlled design assessing the effect of a 4‐week SY meditation training on GM density and intrinsic brain activity. We predicted significant longitudinal structural changes associated with the meditative intervention in the fronto‐insular and inferior temporal regions previously highlighted by cross‐sectional studies on expert SY meditators (Hernandez et al., 2015). To avoid a priori assumptions, we adopted a multimodal whole‐brain approach combining voxel‐based morphometry and a blind gICA decomposition of resting‐state *f*MRI data. We assessed intrinsic brain functioning in terms of spectral power of resting‐state networks (RSNs), a measure of the coherence of intranetwork intrinsic activity (maximal for high power spectra at low frequencies). We chose to constrain our analysis of resting‐state evidence to power spectra because, while representing a sensitive measure of individual differences in intranetwork intrinsic brain functioning (Allen et al., 2011), this metric prevents the need of a priori hypotheses of the regions included in the network(s).

## 2 MATERIALS AND METHODS

### 2.1 Subjects

Forty‐five healthy, right‐handed (Oldfield, 1971) monolingual native Italian undergraduate students (14 females and 31 males, mean age = 21.68, *SD *= 1.57, range = 19–25) were recruited at Bocconi University, Milan. Exclusion criteria were MR incompatibility, prior experience with meditation, history of neurological or psychiatric disease, previous or current use of substances, or any psychoactive medications. Participants gave their written informed consent to the experimental procedure, in accordance with the local Ethics Committee. Subjects were randomly assigned to a 4‐week SY training course (meditation group, MG) or to a 4‐week waiting period (control group, CG). Three participants assigned to the MG dropped out due to lack of time (*n* = 1) or failure in attending classes (*n* = 2). The final sample thus included 12 subjects in the MG (2 females, mean age = 21.63, *SD *= 2.02) and 30 subjects in the CG (12 females, mean age = 22.16, *SD *= 1.33) (Table 1). At baseline, subjects completed the short version of the Temperament and Character Inventory (TCI‐56) (Adan, Serra‐Grabulosa, Caci, & Natale, 2009), assessing four temperament (i.e., harm avoidance, novelty seeking, reward dependence, and persistence) and three character (i.e., self‐directedness, cooperativeness, and self‐transcendence) dimensions (Table 1).

**Table 1:** Characteristics of meditation (MG) and control (CG) groups at baseline (mean ± standard deviation)

	MG (*n* = 12)	CG (*n* = 30)	Statistics
Demographics			
Age (years)	21.63 ± 2.02	22.16 ± 1.33	*t*(40) = 0.99, *p* = 0.33
Sex (F/M)	2/10	12/18	*X* ^2^(1) = 2.1, *p* = 0.15
Temperament and character inventory (TCI‐56)			
Harm avoidance	21.08 ± 6.51	22.87 ± 5.08	*t*(40) = 0.94, *p* = 0.35
Novelty seeking	22.75 ± 4.16	21.8 ± 4.2	*t*(40) = 0.98, *p* = 0.5
Reward Dependence	27.58 ± 7.14	29.3 ± 5.56	*t*(40) = 0.84, *p* = 0.4
Persistence	28 ± 3.64	28.73 ± 5.1	*t*(40) = 0.45, *p* = 0.65
Self‐directedness	28.83 ± 5.5	29.7 ± 4.9	*t*(40) = 0.51, *p* = 0.6
Cooperativeness	27.75 ± 3.47	29.93 ± 4.7	*t*(40) = 1.4, *p* = 0.15
Self‐transcendence	19.42 ± 8.7	22.57 ± 7.98	*t*(40) = 1.25, *p* = 0.27

### 2.2 Training protocol and behavioral statistical analyses

The SY meditation training consisted of four one‐hour sessions per week over 4 consecutive weeks, that is, a total of 16 hr. The overall duration and schedule of the course was planned based on previous evidence on the behavioral effects of short‐term meditation training (Chung, Brooks, Rai, Balk, & Rai, 2012; Fox et al., 1995; Holzel et al., 2011). Participants received guided meditation instructions and joined group activities designed to promote the state of mental silence, known also as thoughtless awareness, held by an instructor with over 25 years (approximately 2,700 hr) of teaching experience. Each hour of daily training was divided into 10–15 min of theoretical lectures, introducing each time a new subject, followed by 45–50 min of meditative practice experience, including various workshop techniques (for further details, see (Manocha, 2014)). No other commitments, including home practice, were required. In the MG, the effect of SY training on subjective well‐being was assessed via non parametric analyses (Wilcoxon signed‐rank test) comparing pre‐ and post‐training scores obtained in a questionnaire providing a measure of emotional self‐assessment. This questionnaire, designed ad hoc based on standardized models (Crawford & Henry, 2004), included 19 questions based on a 10‐level Likert scale assessing general well‐being, subjective experience directly related to meditation (i.e., thoughtless awareness and vibration), as well as the presence of negative (e.g., anger and fatigue) and positive (e.g., joy and peace) emotional states. Despite the existence of validated questionnaires for mood state evaluation, we opted for an ad hoc questionnaire as we aimed at measuring the subjective experiences specifically related to Sahaja Yoga meditation.

### 2.3 MRI data acquisition

Our longitudinal study design entailed two time points for each group, in which we collected anatomical images for voxel‐based morphometry (VBM) analyses of GM density and resting‐state functional images for analyses of intrinsic brain activity. We used a 3 Tesla Philips Achieva scanner (Philips Medical Systems, Best, NL), equipped with an 8‐channel sense head coil (sense reduction factor = 2), to collect anatomical T1‐weighted (150 slices, TR = 600 ms, TE = 20 ms, slice thickness = 1 mm, in‐plane resolution = 1 × 1 mm) and functional T2*‐weighted (gradient‐echo, echo‐planar pulse sequence, 37 continuous ascending transverse slices covering the whole brain, tilted 30° downward with respect to the bicommissural line to reduce susceptibility artifacts in orbitofrontal regions; TR = 2,000 ms, TE = 30 ms, flip angle = 85°, FOV = 192 × 192 mm, slice thickness = 3.7 mm, interslice gap = 0.55 mm, in‐plane resolution = 2 × 2 mm) images. The resting‐state scan was preceded by six “dummy” functional volumes, which were automatically discarded, covering the amount of time needed to allow for T1 equilibration effects. Participants were positioned comfortably on the scanner bed and fitted with soft earplugs; foam pads were used to minimize head movement. They were instructed to lie quietly with their eyes open and stare passively at a foveally presented gray fixation cross to facilitate network delineation (Van Dijk et al., 2010).

### 2.4 VBM data preprocessing and analyses

We performed data preprocessing with the VBM8 toolbox (https://dbm.neuro.uni-jena.de/vbm/), an extension of the SPM8 software (https://www.fil.ion.ucl.ac.uk/spm/), running on MATLAB v7.4 (MathWorks, Inc., Sherborn, MA). VBM preprocessing of the T1‐weighted images included bias correction for field‐intensity inhomogeneities, coregistration of the second image to the baseline image, spatial normalization of the images to a standardized anatomical space, segmentation of the GM component from the normalized images, and smoothing of the normalized GM maps with an 8‐mm full‐width half‐maximum (FWHM) Gaussian kernel. Preliminary analyses based on a two‐sample *t* test excluded statistically significant baseline group differences. The effect of meditation training was assessed with a repeated‐measures ANOVA (i.e., flexible factorial model in SPM8) with time and group as within‐ and between‐subject factors, respectively. We tested an interaction between time and group using a statistical threshold of *p* < 0.05 FWE‐corrected at the cluster level (*p* < 0.001 uncorrected at the voxel level). We localized the clusters showing significant effects using the cytoarchitectonical mapping implemented in the SPM Anatomy Toolbox v 2.2c (Eickhoff et al., 2005). To assess the relationship between GM density and subjective well‐being, we performed non parametric correlations (i.e., Spearman's ρ) between post‐treatment scores obtained at the emotional self‐assessment questionnaire and the average GM values extracted from the significant clusters highlighted by interaction analyses.

### 2.5 rs‐fMRI data preprocessing

Image preprocessing was performed using SPM8 (https://www.fil.ion.ucl.ac.uk/spm), implemented in MATLAB v7.4 (MathWorks, Inc., Sherborn, MA). The 150 volumes from each subject underwent a standard preprocessing including slice‐timing correction with the middle slice in time as a reference, spatial realignment to the first volume and unwarping, spatial normalization into the standard Montreal Neurological Institute (MNI) space (Friston et al., 2002), and resampling in 2 × 2 × 2 mm**^3^** voxels, as well as spatial smoothing with a 8‐mm full‐width half‐maximum (FWHM) isotropic Gaussian kernel. We then applied a procedure of “intensity normalization,” converting the time series of each voxel to percent signal change units, to improve the accuracy and test–retest reliability of the output components of the subsequent independent component analysis (ICA) (Allen, Erhardt, Eichele, Mayer, & Calhoun, 2010; Allen et al., 2011). We used the Motion Fingerprint toolbox.

(https://www.medizin.uni-tuebingen.de/kinder/en/research/neuroimaging/software/) to compute, for each subject, a comprehensive indicator of scan‐to‐scan head motion. An ANOVA including this measure as dependent variable highlighted no significant main effect of group (*F*[1,40] = 0.001, *p* = 0.98) or time point (*F*[1,40] = 2.81, *p* = 0.101), nor a significant interaction between group and time point (*F*[1,40] = 0.48, *p* = 0.494). We also assessed the quality and consistency of spatial normalization across subjects by computing the spatial correlation between the SPM EPI template and both the unsmoothed and smoothed mean images computed from the series of normalized and realigned functional volumes. An ANOVA with such correlation index as dependent variable highlighted no significant main effect of group (smoothed: *F*[1,40] = 1.005, *p* = 0.322; unsmoothed: *F*[1,40] = 0.14, *p* = 0.71), time point (smoothed: *F*[1,40] = 0.08, *p* = 0.78; unsmoothed: *F*[1,40] = 0.23, *p* = 0.635), or interaction between group and time point (smoothed: *F*[1,40] = 0.001, *p* = 0.97; unsmoothed: *F*[1,40] = 0.27, *p* = 0.605).

### 2.6 Group Independent Component Analysis (gICA)

We used multivariate spatial gICA, as implemented in the GIFT toolbox (https://icatb.sourceforge.net;(Allen et al., 2011; Calhoun, Adali, Pearlson, & Pekar, 2001a), to extract temporally coherent and maximally independent spatial sources, that is, functional networks or “spatial maps,” from resting‐state time courses. The independent component analysis was preceded by a data‐reduction stage, based on a principal component analysis (PCA) retaining 100 principal components from single subjects’ time courses (Erhardt et al., 2011). Subsequent gICA retained 75 components through a neural network algorithm (Infomax) that attempts to minimize the mutual information of the network outputs to identify naturally grouping and maximally independent sources (Bell & Sejnowski, 1995). ICA was repeated 250 times in Icasso (https://www.cis.hut.fi/projects/icasso). The resulting components were clustered to ensure the consistency and reliability of the decomposition, which are quantified using a quality index Iq ranging from 0 to 1, reflecting the difference between intracluster and extracluster similarity (Himberg, Hyvarinen, & Esposito, 2004). Subject‐specific spatial maps and time courses were estimated with GICA3 back‐reconstruction (Calhoun, Adali, Pearlson, & Pekar, 2001b; Erhardt et al., 2011).

### 2.7 Resting‐state networks selection and identification

Alongside the Iq index, we used the spectral characteristics of component time courses to discriminate reliable resting‐state networks (RSNs) from physiological artifacts. Based on the notion that normal resting‐state time courses are dominated by slow (i.e., low frequency) fluctuations (Cordes et al., 2000), we evaluate “dynamic range” (i.e., the difference between peak spectral power and minimum power at frequencies to the right of the peak) and “low‐frequency‐to‐high‐frequency power ratio” (i.e., the ratio of the integral of spectral power below 0.1 Hz to the integral of power between 0.15 and 0.25 Hz) (Allen et al., 2011). In addition, the aggregate spatial maps underwent a visual inspection by three independent raters, based on expectations that they should involve GM rather than known ventricular, vascular, susceptibility or motion‐related artifacts. Each rater scored spatial maps by assigning them to one of three possible classes, that is, definite artifact (0), mixed (1), or genuine resting‐state component (2). We retained only the components assigned to the latter class by all raters. The two spectral characteristics, alongside an Iq > 0.8 and the visual inspection of the aggregate spatial maps, led to select a subset of 41 out of 75 components as genuine RSNs. We anatomically labeled the selected RSNs based on the largest spatial correlation between the spatial maps of each component and the RSNs template provided with the GIFT toolbox. A refined labeling of the components showing a significant time‐by‐group interaction (see below) was performed using the cytoarchitectonic maps implemented in the SPM Anatomy toolbox v2.2c (Eickhoff et al., 2005).

### 2.8 RSN statistical analyses

In subsequent statistical analyses, we considered the power spectra of RSN time course, representing the contribution of specific frequency bins to the slow synchronous fluctuations of the BOLD signal. This metric reflects the degree of intranetwork coherent activity, which is maximal at the typical resting‐state frequencies below 0.1 Hz, while a relative shift toward higher frequencies has been proposed to reflect altered intranetwork connectivity in physiological aging (Allen et al., 2011) and neurological diseases (Caminiti et al., 2015). We estimated spectra on the detrended subject‐specific time courses, after removal of the mean, slope, and period π and 2π sines and cosines over each time course.

In the main analysis, we assessed the effects of group (i.e., MG and CG), time (i.e., pretreatment and post‐treatment), as well as the interaction between group and time, by using a backward multivariate model selection strategy for the outcome variable (i.e., power spectra). This procedure first employs a multivariate analysis of covariance (MANCOVA) to select which factors explain variability in the outcome measure. Then, univariate tests corrected for multiple comparisons are carried out on a reduced design matrix (thus decreasing the number of statistical tests performed), to highlight the direction and strength of the relationship between retained factors and power spectra. As for VBM analyses, we first performed preliminary analyses comparing power spectra metrics in MG and CG to exclude significant baseline group differences. The effects of interest in the design matrix were group, time, and their interaction, as well as two nuisance predictors reflecting the quality of spatial normalization (of smoothed normalized images) and average scan‐to‐scan head motion. As previously discussed, we also aimed to test a relationship between changes in the metrics of intrinsic brain activity and subjective well‐being. To this purpose, we then used non parametric statistics (i.e., Spearman's ρ) to assess a correlation between power spectra in the frequency bins displaying a significant time‐by‐group interaction and the scores obtained at the questionnaire of emotional self‐assessment in the post‐treatment stage.

To investigate a connection between the functional (rs‐fMRI) and structural (morphometric) levels of analysis, we also tested whether meditation training was associated with a significant increase of GM density in the network(s) showing an interactive effect of group and time on resting‐state power spectra. To this purpose, we first used the SPM toolbox Marsbar (https://marsbar.sourceforge.net) to convert the gICA maps showing a significant interactive effect into binary regions of interests (ROIs). Then, we used the toolbox REX (https://web.mit.edu/swg/software.htm) to extract, from the resulting masks, average GM density values for subsequent off‐line statistical analyses. For each resulting map, we used a mixed ANOVA to compare GM density in meditators vs controls before and after treatment.

All the reported results survived a statistical threshold of *p* < 0.05 corrected for multiple comparisons using false discovery rate (FDR; (Genovese, Lazar, & Nichols, 2016)).

## 3 RESULTS

### 3.1 Behavioral results

The final samples did not differ significantly in mean age (*t*[40] = 0.99, *p* = 0.33) or gender (*X* ^2^[1] = 2.1, *p* = 0.15). In addition, no significant differences were found between MG and CG in any of the TCI‐56 subscales (Table 1). Due to the small sample size, we performed preliminary analyses on behavioral variables with a parametric distribution, which excluded the presence of outliers among subjects. All MG subjects included in the final sample completed the 16 hr of scheduled training. The within‐group analysis on the scores of emotional self‐assessment revealed a significant increase in general well‐being (*Z* = 3.06, *p* = 0.002), as well as a significant reduction in fatigue (*Z* = 2.67, *p* = 0.007) and dissatisfaction (*Z* = 1.91, *p* = 0.05), after the meditative training (Table 2).

**Table 2:** Training‐related effects on well‐being and emotional self‐assessment

	Meditation group		Statistics
	Pretraining	Post‐training	
Emotional self‐assessment			
General well‐being	5.33 (0.98)	7 (0.739)	***Z* = 3.06, *p* = 0.002**
Thoughtless awareness	6.48 (1.76)	7.49 (1.35)	*Z* = 1.34, *p* = 0.18
Vibration cool/warm	7.13 (0.84)	7.54 (1.28)	*Z* = 0.71, *p* = 0.48
Nervousness	2.97 (1.74)	2.11 (1.39)	*Z* = 1.61, *p* = 0.18
Fatigue	4.65 (1.73)	3.18 (2.20)	***Z* = 2.67, *p* = 0.007**
Anger	2.88 (2.39)	1.76 (1.53)	*Z* = 1.16, *p* = 0.25
Fear	1.10 (087)	1.09 (1.04)	*Z* = 0.35, *p* = 0.72
Dissatisfaction	3.79 (2.56)	2.38 (1.57)	***Z* = 1.91, *p* = 0.05**
Guilt	2.82 (2.34)	2.90 (1.91)	*Z* = 0.26, *p* = 0.79
Joy	6.39 (1.33)	6.12 (1.69)	*Z* = 0.84, *p* = 0.40
Peace	5.82 (2.51)	6.74 (1.76)	*Z* = 1.1, *p* = 0.27
Satisfaction	4.68 (2.39)	6.11 (1.49)	*Z* = 1.69, *p* = 0.09
Compassion	4.47 (2.69)	5.44 (1.76)	*Z* = 1.88, *p* = 0.06
Self‐confidence	5.51 (2.39)	6.38 (1.73)	*Z* = 1.41, *p* = 0.15
Aware of attention	5.05 (2.40)	5.77 (1.94)	*Z* = 0.18, *p* = 0.86
In control of attention	6.24 (2.07)	6.21 (1.63)	*Z* = 0.039, *p* = 0.97
Creativity	5.01 (2.25)	5.38 (1.79)	*Z* = 0.47, *p* = 0.63
Open to change	7.33 (1.17)	7.68 (1.04)	*Z* = 1.38, *p* = 0.17
Openness with others	5.19 (2.32)	5.47 (1.73)	*Z* = 0.94, *p* = 0.34

Note

Scores obtained at the questionnaire of emotional self‐assessment by the meditation group before and after training. The mean and standard deviation (in brackets) are reported for each variable. Significant effects are depicted in bold font.

### 3.2 VBM results

**Figure 1:**
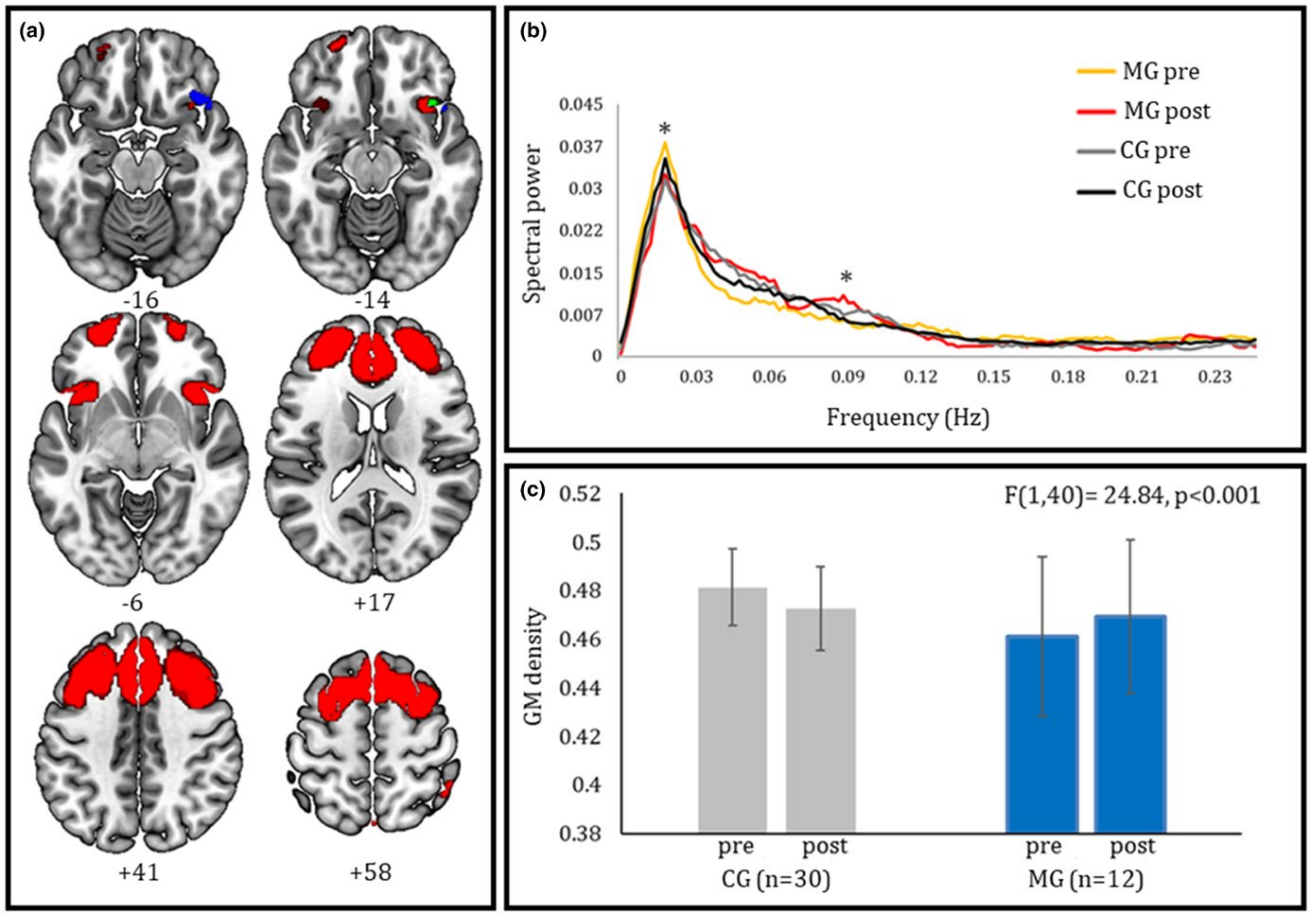
(a) Spatial contiguity between the right inferior frontal clusters showing increased GM density (in blue) and a modulation of coherent activity (in red) after meditation training. The overlap between morphometric and resting‐state data is shown in green. (b) Spectral power of intrinsic activity in the executive control network, providing a measure of the contribution of each frequency bin (between 0 and 0.25 Hz) to the fluctuations of BOLD signal at rest (asterisks indicate the frequency bins displaying a significant effect in time‐by‐group interaction). Meditators, compared with non meditators, display a reduction of power at ultra‐low frequencies, and an increase at low–middle frequencies, after training. (c) Average GM density in the cluster resulting from VBM interaction analysis for the two time points of both training (MG) and control (CG) groups (error bars depict standard deviations). Meditators, compared with non meditators, display a significant increase of GM density with training in the right fronto‐insular cluster depicted in green color in panel A

We found no significant group difference in GM density at baseline. Time‐by‐group interaction analyses showed, in meditators compared with control subjects, a significant increase after training of GM density in a cluster encompassing the right inferior frontal gyrus (rIFG) (*k* = 336, local maxima MNI coordinates *x*;*y*;*z* = 40;26;−21) (Figure 1a,c). A 2 × 2 repeated‐measures ANOVA confirmed a significant “time × group” interaction (*F*[1,40] = 24.84, *p* < 0.001), with no significant effects of time (*F*[1,40] = 0.059, *p* = 0.809) and group (*F*[1,40] = 2.58, *p* = 0.116) alone. Moreover, non parametric correlation analyses showed a positive correlation between average GM density in this cluster and general well‐being after training (Spearman's ρ = 0.615, *p* = 0.03).

### 3.3 RSN networks

The visual inspection of the spatial maps, alongside the analysis of spectral profiles, led to select 41 components which largely overlapped the RSNs previously described (e.g. (Allen et al., 2011; Caminiti et al., 2015)). Namely, they involved the anterior and posterior components of the default mode network (Buckner, Andrews‐Hanna, & Schacter, 2008), as well as the anterior and posterior portions of the salience network (Seeley et al., 2007), alongside the dorsal attentional network (Corbetta & Shulman, 2002). Distinct frontal networks included regions belonging to the fronto‐parietal executive control network (Vincent, Kahn, Snyder, Raichle, & Buckner, 2008), as well as fronto‐temporal and fronto‐limbic regions associated with, respectively, linguistic and affective processing. Finally, we identified components representing both primary and higher‐level visual networks, as well as the sensorimotor and auditory networks.

### 3.4 Group effects on power spectra

As for VBM analyses, we found no significant baseline group difference in terms of resting‐state metrics. A time‐by‐group interaction on the level of coherent activity (i.e., power spectra) was assessed in all the retained components. We found a significant interaction in the frontal sector of the fronto‐parietal “executive control” network (Vincent et al., 2008), involving the bilateral dorsolateral and dorsomedial prefrontal cortex, the dorsal anterior cingulate cortex (dACC), and bilateral fronto‐insular cortex (Figure 1a). Within this component, the MG displayed compared with CG decreased power at ultra‐low frequencies (<0.02 Hz) and increased power in the typical low–middle frequencies (<0.1 Hz) after training (Figure 1b). In addition, higher post‐training well‐being scores were reflected in lower power spectra at ultra‐low frequencies (<0.02 Hz) within the same network (Table 3).

**Table 3:** Relationship between well‐being and neural metrics after training

	Power spectra				GM density
	Ultra‐low frequencies (<0.02 Hz)				
	Bin 7	Bin 8	Bin 9	Bin 10	
General well‐being	*r* = −0.71 *p* < 0.01	*r* = −0.72* p* < 0.01	*r* = −0.54* p* = 0.07	*r* = −0.51 * p* = 0.08	*r* = 0.615 *p* < 0.05
	Low frequencies (<0.1 Hz)				
	Bin 48				
Fatigue	*r* = −0.56 *p* = 0.05				

Note

Significant correlations in the meditation group between resting‐state metrics, GM density, and scores at the questionnaire of self‐emotional assessment after training. GM: Gray matter.

There was no significant effect of training on GM density in the whole component highlighted by the above resting‐state findings. However, when assessing its different subclusters separately we observed that the GM increase from pre‐ to post‐training was larger, in the MG than CG (t[40] = 2.22, *p* = 0.03), in a right inferior fronto‐insular region adjacent to that highlighted by VBM analyses (Figure 1a).

## 4 DISCUSSION

Available evidence suggests that distinct meditation practices reflect in a differential involvement of neural systems associated with selective and sustained attention vs monitoring, vigilance, and representation of internal bodily states (Lutz et al., 2008). In particular, SY meditation has received growing attention in the last years due to its beneficial effects on different psycho‐physiological variables (Aftanas & Golocheikine, 2001; Chung, Brooks, Rai, Balk, & Rai, 2012). While previous related studies have taken a cross‐sectional approach to assess the long‐term effects of this type of meditation (Aftanas & Golocheikine, 2001; Aftanas & Golosheikin, 2003; Hernandez et al., 2007, 2015; Reva et al., 2014), here we employed a longitudinal randomized controlled approach to investigate a causal relationship between meditation practice and cortical reorganization, as indexed by brain structure and spontaneous activity.

Subjects participating in the meditation intervention displayed a significant improvement in self‐perceived general well‐being after training. In addition, compared with control subjects they also showed a significant change in brain structure and intrinsic activity in inferior fronto‐insular regions associated with executive control, and previously highlighted by cross‐sectional studies on the neural effects of long‐term SY meditation (Hernandez et al., 2015).

With regard to the functional modifications observed in intrinsic brain functioning, we provide novel evidence of a causal connection between SY practice and significant changes in the distribution of low–middle frequencies of resting‐state activity. A significant time‐by‐group interaction on spectral power highlighted, in meditators vs controls, a training‐related remodeling of the contribution of slow frequencies in the anterior component of the fronto‐parietal “executive control” network. The anterior section of this network, comprising the dorsolateral and dorsomedial prefrontal cortex, the dACC, as well as the inferior fronto‐insular cortex bilaterally, is thought to be involved in goal‐directed behavior (Spreng, Stevens, Chamberlain, Gilmore, & Schacter, 2010; Sutherland, McHugh, Pariyadath, & Stein, 2012; Vincent et al., 2008). This set of regions, which is commonly recruited by tasks requiring controlled information processing (Dosenbach et al., 2007), has been recently proposed as a “superordinate” cognitive control network, recruited across different executive domains including flexibility, working memory, initiation, and inhibition (Niendam et al., 2012). Available evidence suggests that specific nodes of this network play a key role in orienting the attentional focus to the external vs internal environment (Spreng, Sepulcre, Turner, Stevens, & Schacter, 2013). Among these regions, the right inferior fronto‐insular cortex acts as a critical node in suppressing default activity and re‐allocating attentional resources to salient events via the engagement of dorsal attention networks (Sridharan, Levitin, & Menon, 2008). The role of this region in regulating the relative engagement of default mode vs dorsal attentional networks—based on the salience of stimuli with respect to behavioral goals (Menon & Uddin, 2010)—accounts for previous evidence relating this region, in conjunction with dACC, to self‐regulation (Craig, 2009; Seeley et al., 2007) as well as initiation, maintenance, and adjustment of attentional control (Dosenbach et al., 2007; Dosenbach, Fair, Cohen, Schlaggar, & Petersen, 2008). This concept fits with the demands posed by SY meditation, aiming to obtain a state of mental silence in which the attention is focused on the present moment. Changes of intranetwork coherent activity in such a control network may support the attempt to resist narrative mind‐wandering (Mason et al., 2007), allowing to maintain in working memory the awareness of the present moment and monitor one's body state.

This hypothesis fits with neuroimaging evidence on other types of short‐term meditation interventions, sharing with the SY meditation an open monitoring approach aiming to develop the capacity for mindfulness (i.e., awareness of present‐moment experiences with a compassionate, non judgmental, stance). Across different studies, such interventions resulted in stronger intensity of activation (Holzel et al., 2011) and efficiency (Xue, Tang, & Posner, 2011) in functional networks involving the dACC, possibly reflecting higher cognitive control and improved suppression of distracting events. Similar studies highlighted a stronger recruitment of the dorsolateral prefrontal cortex alongside the right insula after training (Farb et al., 2014), which have been associated with an increased awareness of momentary self‐reference. The latter result was also associated with reduced activity in key nodes of the default mode network, related to extended and narrative self‐reference, which may reflect the enduring absence of narrative thought prompted by meditation (Mason et al., 2007). It is noteworthy that one of the few previous studies investigating the neuro‐functional correlates of mental silence in SY meditation experts has shown a significant relationship between thoughtless awareness and the activation in the middle/superior temporal and right fronto‐insular cortex, with the latter being directly related to the subjective depth of the mental silence experience (Hernandez et al., 2007). Overall, these data consistently point to the role of the executive control network in orienting attention to the external or internal environment as a prerequisite of mental silence (i.e., interruption of unnecessary mental activity). Interestingly, the same authors have also provided evidence of increased GM in fronto‐insular cortex in long‐term SY meditators (Hernandez et al., 2015).

These findings, highlighting the effects of SY meditation on the functional and structural properties of the executive control network, fit with the present evidence of morphometric changes, after meditation training, in the same right fronto‐insular region described above. While analogous morphometric changes resulting from different short‐term trainings have been previously reported (e.g. after a visuomotor training in (Draganski et al., 2004)), their functional meaning is still debated. Alongside the functional changes in intrinsic brain activity previously discussed, the increase of GM density in the right inferior fronto‐insular cortex with meditation training may underpin the beneficial effects of SY on the efficiency of cognitive control and attentional allocation, key processes for monitoring the moment‐to‐moment experience and constraining narrative self‐reference. Importantly, both these brain changes appear to parallel the beneficial effects of SY training on perceived well‐being, as indicated by the significant correlations with the scores obtained at the questionnaire of emotional self‐assessment after training.

While such evidence highlights the potential for brain plasticity beyond the developmental stages, however, the current spatial resolution of MRI data does not allow to distinguish different microstructural processes which may underlie VBM results, for example, dendritic arborization vs axon remodeling (Mietchen & Gaser, 2009). Previous literature suggests that functional and structural plasticity might depend on the balance between processes promoting molecular flexibility (e.g., Hebbian plasticity) and stability (e.g., homeostatic plasticity) (Yin & Yuan, 2014), but the interaction between these mechanisms, the subsequent large‐scale consequences on brain function and structure, and the behavioral response remain to be clarified (Hamaide, De Groof, & Van der Linden, 2004).

A limitation of this study is represented by the presence of a passive control group. However, this choice was driven by concerns regarding the cognitive and behavioral processes which could, or could not, be controlled in any “active” control procedure. Moreover, the lack of significant differences between active and passive control groups in previous studies (Chooi & Thompson, 2012; Colom et al., 2016) led us to adopt the most conservative procedure. Moreover, the use of an ad hoc questionnaire, rather than a validated one, might limit the robustness of the behavioral results. However, to the best of our knowledge, no formal measurements are currently available to assess the subjective experiences specifically related to SY meditation and future studies could benefit from building upon the questionnaire here proposed. Another possible limitation is represented by the relatively small sample size of the active group, partially due to dropout. Although preliminary analyses confirmed the intergroup homogeneity concerning the variables of interest (thus reducing the impact of potential sources of error), additional studies with larger sample size are needed to control for the potential impact of intersubject variability. Finally, the present evidence on meditation‐related effects on spectral power of intrinsic intranetwork brain activity should be complemented by a system‐level description of the associated changes in internetwork functional connectivity. Such evidence will likely help refining an interpretation of the present evidence on spectral power, which still remains an open issue.

In conclusion, this is the first longitudinal randomized controlled study examining a causal relationship between SY meditation practice and cortical reorganization, as indexed by brain structure and spontaneous activity. Evidence from both brain morphometry and resting‐state activity consistently pointed to the right inferior fronto‐insular sector of the executive control network as a crucial target of SY meditation, modulating brain activity and structure in this key node of the networks underlying attentional control. A significant increase of GM density in the fronto‐insular cortex, a change in coherent activity in the whole network, as well as the correlations with well‐being measures, consistently suggest a causal link between SY practice and the development of some of the core experiential correlates of SY meditation, such as the individual capacity to access positive affect states and long‐term emotional well‐being, together with the capacity to consciously modulate internal and external attention control mechanisms. These mechanisms may thus underlie the enhanced ability to reach a state of thoughtless awareness, which can be connected to reduced unnecessary mental activity and more efficient use of cognitive skills. Our results confirm and extend previous cross‐sectional evidence on the structural (Hernandez et al., 2015) and functional (Hernandez et al., 2007) correlates of SY in expert meditators. Unlike previous studies, however, here we show a persistent effect of meditation outside the formal practice. In line with previous evidence of significant differences between expert meditators and controls in the electrophysiological spectral profile at rest (Lutz, Greischar, Rawlings, Ricard, & Davidson, 2004), our results suggest a potential broad impact of meditative practice on neural organization, in turn reflecting on other outcomes related to health and well‐being (Muehsam et al., 2017).

## CONFLICT OF INTEREST

None declared.
